# SAPHO syndrome with membranous nephropathy and analgesic-induced nephropathy: a case report

**DOI:** 10.3389/fimmu.2026.1784305

**Published:** 2026-06-02

**Authors:** Yuxin Qiao, Zuo Wang, Guohua Li, Duo Zhang

**Affiliations:** 1 Clinical College of Traditional Chinese Medicine, Inner Mongolia Medical University, Hohhot, China; 2 Department of Rheumatology and Immunology, Inner Mongolia Autonomous Region Hospital of Traditional Chinese Medicine, Hohhot, China; 3 Department of Nephrology, Inner Mongolia Autonomous Region Hospital of Traditional Chinese Medicine, Hohhot, China

**Keywords:** analgesic nephropathy, case report, headache, membranous nephropathy, SAPHO syndrome

## Abstract

SAPHO syndrome is a rare autoimmune disorder, and the occurrence of renal involvement in this condition is even more uncommon. We report the case of a 43-year-old male patient with SAPHO syndrome, who initially presented with palmoplantar pustulosis and pain in the chest wall and sternoclavicular joints, accompanied by proteinuria and microscopic hematuria. He was admitted to the hospital due to headache, lower limb edema, and elevated serum creatinine. Renal biopsy revealed stage II membranous nephropathy combined with analgesic nephropathy. Following treatment, his symptoms resolved, and at one-year follow-up, proteinuria had nearly achieved clinical complete remission, with stable renal function.

## 1 Introduction

SAPHO syndrome (synovitis, acne, pustulosis, hyperostosis, and osteitis) is a rare chronic inflammatory disorder primarily affecting the bones, joints, and skin, often leading to chronic pain, impaired joint function, and reduced quality of life. The disease predominantly affects women aged 30–50 years (1), with a prevalence of less than 1:10,000 (2). The pathogenesis of SAPHO syndrome has not been fully elucidated, but it is believed to involve a combination of genetic, infectious, and immune factors, resulting in activation of both innate and cell-mediated immune responses (3). Clinical manifestations are highly variable, and the lack of specific diagnostic markers often leads to misdiagnosis or underdiagnosis. Renal involvement in SAPHO syndrome is extremely rare. A literature search of the CNKI, Chinese Medical Journals, and PUBMED databases using the keywords “SAPHO syndrome, renal, kidney, nephropathy, nephritis, proteinuria” identified only six reported cases of SAPHO syndrome with renal impairment, presenting as AA amyloidosis, IgA nephropathy, membranous nephropathy, hydronephrosis, or renal failure, suggesting diverse patterns of kidney injury. Here, we report a case of SAPHO syndrome complicated by stage II membranous nephropathy and analgesic nephropathy secondary to excessive use of painkillers for severe headache, and we analyze the associated clinical features.

## 2 Case presentation

A 43-year-old male was admitted on June 18, 2024, with a chief complaint of joint pain, skin pustules, frothy urine for more than six years, and 47 days of headache accompanied by swelling and elevated serum creatinine for 16 days. The patient first developed pain in the chest wall and sternoclavicular joints without obvious triggers in September 2017, which was partially relieved with oral loxoprofen. In October 2017, he developed bilateral palmoplantar pustulosis and rashes on the extremities. By December 2017, his symptoms worsened, accompanied by increased urinary foaming. Urinalysis showed proteinuria (2+), microscopic hematuria (2+), and red blood cells 19/μL; 24-hour urine protein quantification was 0.51 g/24 h. Skin biopsy confirmed palmoplantar pustulosis (**Figure 1**), but no further intervention was undertaken. On May 2, 2024, the patient experienced severe headache without an apparent trigger, predominantly a throbbing sensation in the vertex and occipital region, radiating to the bilateral temporal and facial areas. Laboratory tests revealed persistent proteinuria (2+) and hematuria (3+); elevated serum γ-glutamyl transferase (173 U/L); hypoalbuminemia (28 g/L); interleukin-1β 4.00 pg/mL, interleukin-2 receptor 783.86 U/mL, interleukin-6 68.15 pg/mL, interleukin-8 67.43 pg/mL, and TNF-α 1.32 pg/mL (**Table 1**). Cervical spine CT and MRI, cranial CT and MRI, contrast-enhanced skull base MRI, and lumbar puncture with cerebrospinal fluid analysis were unremarkable. Whole-body bone scintigraphy demonstrated diffuse radiotracer uptake around the manubrium and bilateral first costosternal joints, showing a typical “bullhead sign” (**Figure 2**). CT revealed bone destruction of the sternum (**Figure 3**). Based on these findings, the patient was diagnosed with SAPHO syndrome, with headache under further evaluation and possible nephrotic syndrome. Between May 2 and May 17, the patient self-administered a cumulative total of 300 tablets of Somitop, 72 sustained-release capsules of ibuprofen, and 24 tablets of acetaminophen/oxycodone, achieving only temporary pain relief for a few hours before recurrent exacerbation. Subsequent standardized treatment with mannitol, sodium aescinate, and acetaminophen/oxycodone for 10 days produced minimal improvement. On June 2, he developed fever with temperatures ranging from 37.3 °C to 37.8 °C, accompanied by swelling in both lower extremities. The patient was admitted on June 18. Since May 2, his body weight had decreased by 10 kg (13.5%). He denied any family history of similar conditions. Physical examination revealed generalized skin rashes, most prominent over the lumbosacral region, with roughened skin on the bilateral palms and soles (**Figure 4**), as well as moderate pitting swelling of both lower limbs. Laboratory tests revealed proteinuria (3+) and hematuria (3+), with 135 red blood cells/μL and 24-hour urine protein quantification of 12.5 g/24 h. Complete blood count showed a white blood cell count of 12.99 × 10^9^/L with 90.7% neutrophils, hemoglobin 114 g/L, and platelets 387 × 10^9^/L. Serum creatinine was 120.9 μmol/L, and serum albumin was 27.2 g/L. C-reactive protein was 20 mg/L, and erythrocyte sedimentation rate was 105 mm/h. Tests for systemic lupus erythematosus and anti-phospholipase A2 receptor antibodies were negative. Color Doppler ultrasonography demonstrated enlarged kidneys with diffuse lesions. Renal biopsy (**Figure 5**) revealed stage II membranous nephropathy complicated by acute analgesic nephropathy. The patient received intravenous cefuroxime sodium 3 g/day for 5 days, intravenous methylprednisolone 40 mg/day, and oral mycophenolate mofetil 1.5 g/day. After 14 days of treatment, his symptoms resolved, and serum creatinine decreased to 64.2 μmol/L. Upon discharge, the patient continued regular medications with gradual tapering. Follow-up color Doppler ultrasonography in November of the same year showed normalization of kidney size, and long-term monitoring revealed 24-hour urine protein levels controlled at approximately 0.5 g, with preserved renal function. The timeline of the patient’s clinical course is summarized (**Figure 6**), and detailed laboratory findings are presented (**Table 2**).

**Figure 1:**
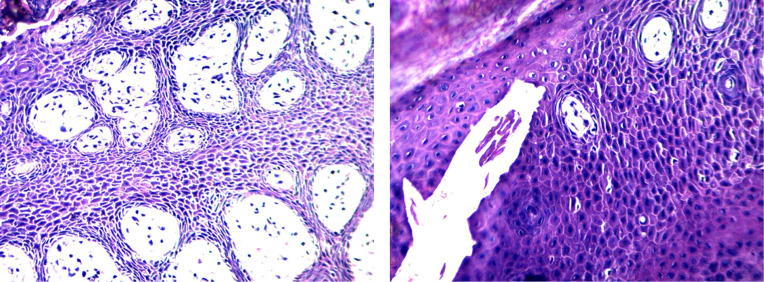
Skin biopsy of the palm (showing a unilocular pustule between prickle cells and inflammatory cell infiltration in the superficial dermal papillae).

**Table 1:** Peripheral blood inflammatory markers and reference ranges.

Date	Test	Result	Reference range
2024-05	IL-6	68.15 pg/mL↑	≤7 pg/mL
	IL-8	67.43 pg/mL↑	≤19.7 pg/mL
	IL-2R	783.86 U/mL↑	≤710 U/mL
	IL-1β	4.00 pg/mL	≤11.2 pg/mL
	TNF-α	1.32 pg/mL	≤5.1 pg/mL

**Figure 2:**
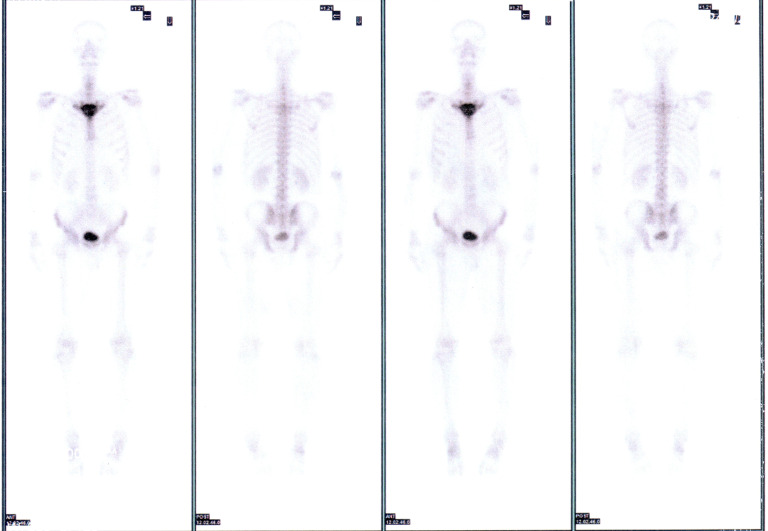
Whole-body bone scan (showing diffuse areas of increased radioactivity around the sternal manubrium and bilateral first sternocostal joints, presenting a typical "bull's head sign").

**Figure 3:**
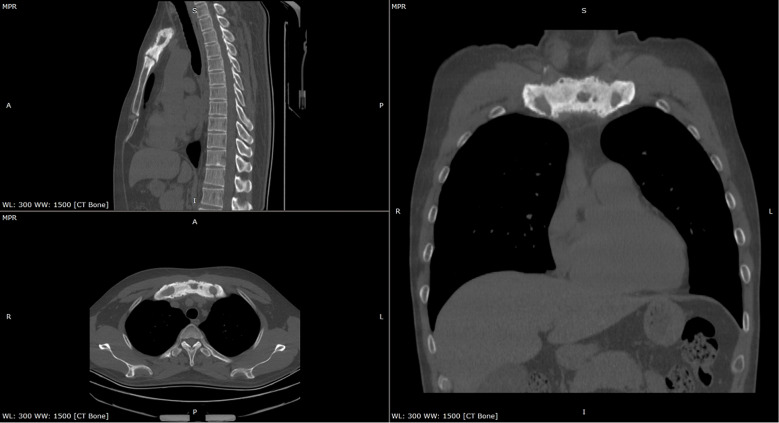
Chest CT (showing destruction of the sternal bone, uneven bone density involving the sternoclavicular joints, and high-density shadows in the surrounding soft tissue with indistinct borders).

**Figure 4:**
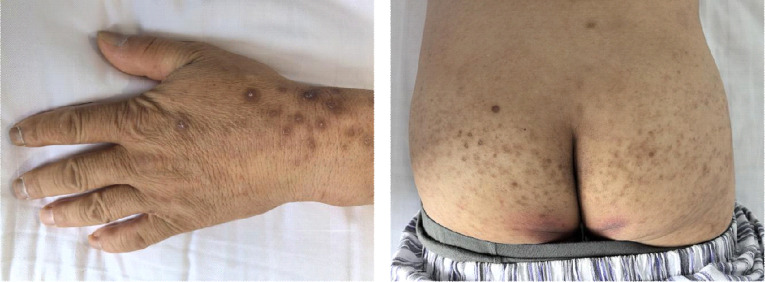
Rashes ranging from millet- to broad bean-sized on the dorsum of the left hand and symmetrical rashes on both buttocks.

**Figure 5:**
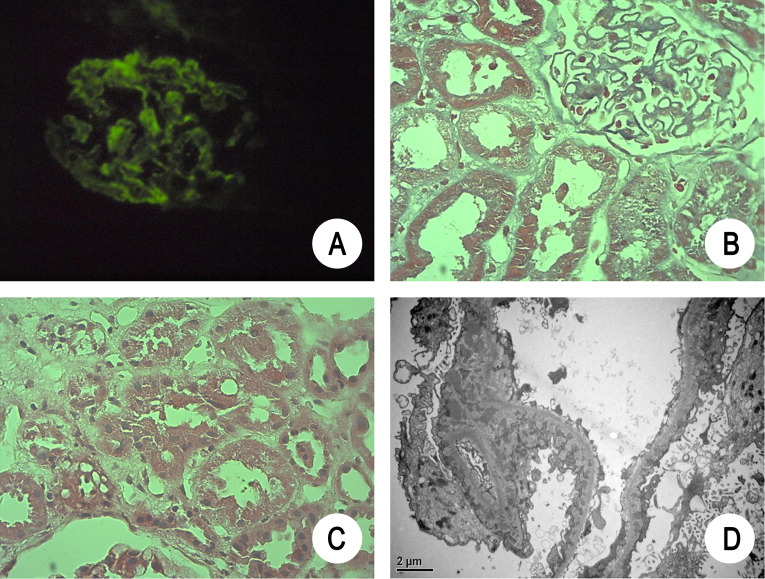
Pathological changes of renal biopsy. [**A** Immunofluorescence (×400) shows IgG 3+ and C3 2+ with granular deposition along the glomerular capillary loops. **B** Light microscopy (Masson staining, ×400) reveals diffuse thickening of the glomerular basement membrane accompanied by numerous spike formations. **C** Light microscopy demonstrates focal atrophy of renal tubules, some showing bare basement membranes; extensive and diffuse edema of the renal interstitium with focal infiltration of lymphocytes and monocytes. **D** Electron microscopy (×5000) shows numerous electron-dense deposits in the subepithelial region of glomerular cells; the basement membrane exhibits spike-like thickening with fusion of podocyte foot processes].

**Figure 6:**
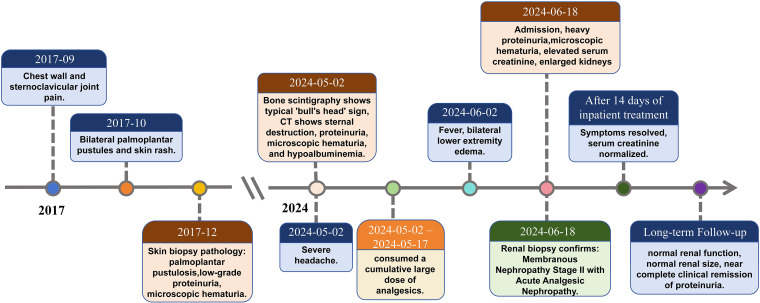
Time course of the individual medical history presented.

**Table 2:** Detailed laboratory test results.

Parameter Time	SCr(μmol/L)	ALB(g/L)	GGT (U/L)	WBC(×10^9^/L)	NEU%	RBC(×10¹²/L)	HGB(g/L)	PLT(×10^9^/L)	U-Pro	U-BLD	U-RBC (μL)	24h-UPro (g)
2017-12	62.0	43.6	28	7.51	63.3	4.69	152	276	2+	2+	19 ↑	0.51 ↑
2024-05	55↓	28↓	173 ↑	10.28 ↑	82.3 ↑	3.85 ↓	125 ↓	430 ↑	2+	3+	24 ↑	-
2024-06	120.9 ↑	27.2 ↓	161.4 ↑	12.99 ↑	90.7 ↑	3.85 ↓	114 ↓	387 ↑	3+	3+	135 ↑	12.5 ↑
2024-07	64.2	21.1 ↓	51	9.41	65.1	4.60	134	219	3+	3+	36↑	-
2024-08	66.4	24.0 ↓	45.9	12.01 ↑	70.8	4.51	138	261	3+	3+	0	-
2025-07	75.0	47.7	31	10.87 ↑	77.7↑	4.62	146	305	-	-	-	0.59 ↑

## 3 Discussion

The diagnosis of SAPHO syndrome is primarily based on the characteristic involvement of bones, joints, and skin, and requires differentiation from other disorders with similar clinical features. For instance, psoriatic arthritis presents with arthritis and pustular psoriasis, with joint involvement typically affecting peripheral joints and the sacroiliac joints. Typical nail changes of psoriasis are often observed, and renal involvement, when present, most commonly manifests as IgA nephropathy (4); Infectious osteomyelitis typically presents with an acute onset, often accompanied by fever and localized redness, swelling, and tenderness, along with systemic toxic symptoms. Imaging may reveal sequestra or abscess formation, and microbiological tests are frequently positive. Additionally, differential diagnoses include malignancies, ankylosing spondylitis, dermatomyositis, acne, and pustulosis. In the present case, after excluding these conditions, the combination of a chronic disease course, palmoplantar pustulosis, the “bullhead sign” on whole-body bone scintigraphy, sternal bone destruction, weight loss, elevated inflammatory markers, and a favorable response to immunosuppressive therapy supports the diagnosis of SAPHO syndrome and reflects active disease.

During the course of disease, the patient developed severe headache, with no obvious abnormalities detected on imaging, which increased the diagnostic complexity. Literature review indicates that headache associated with SAPHO syndrome may result from involvement of the cranial bones and dura mater: in some cases, MRI revealed dural thickening and enhancement, as well as abnormal bone marrow signals in the skull, with biopsy confirming inflammatory infiltration of both bone and dura mater (5, 6); other reports describe initially negative imaging findings, with progression to lytic skull lesions one year later (7); isolated cranial bone lesions without involvement of other bones or joints have also been reported (8). In the present case, no cranial or dural biopsy was performed, and therefore direct histological evidence is lacking. However, inflammatory markers were markedly elevated (e.g., IL-6, IL-8, platelet count, CRP, ESR), multiple analgesics provided minimal relief, and corticosteroid therapy was effective, suggesting that the headache may have originated from an early inflammatory state of the cranial bones or dura mater related to SAPHO syndrome, prior to the development of structural changes.

In this patient, renal injury involved both membranous nephropathy and analgesic nephropathy, both contributing to the progression of kidney damage. Proteinuria gradually progressed to the nephrotic range, accompanied by persistent microscopic hematuria. Anti-PLA2R antibodies were negative, which differs from primary membranous nephropathy, where hematuria is typically minimal and sporadic and anti-PLA2R antibodies are often positive, suggesting secondary membranous nephropathy associated with SAPHO syndrome.

The pathogenesis of SAPHO-associated secondary membranous nephropathy may involve several mechanisms. Exogenous antigens or newly exposed epitopes may abnormally accumulate in the subepithelial region of the glomerular basement membrane, becoming targets for circulating antibodies (9). SAPHO syndrome is characterized by immune dysregulation, leading to excessive neutrophil activation and the release of pro-inflammatory mediators (10). These cytokines can reach the kidney via systemic circulation, inducing local inflammation and further compromising the glomerular filtration barrier. For example, IL-6 can downregulate VE-cadherin expression in glomerular endothelial cells via trans-signaling, increasing endothelial permeability (11), while IL-8 recruits neutrophils through CXCR1/2 receptors, amplifying local inflammatory effects (12). Additionally, the chronic inflammatory microenvironment in SAPHO syndrome may drive aberrant B-cell activation, resulting in the production of pathogenic autoantibodies targeting podocyte antigens. These antibodies may not recognize classical PLA2R antigens but instead specifically target other podocyte-related antigens, such as THSD7A or NELL-1, leading to subepithelial *in situ* immune complex deposition. The deposited immune complexes can activate the complement system, generating C5b-9 membrane attack complexes, which ultimately damage podocytes, causing cytoskeletal remodeling, slit diaphragm disruption, and resultant proteinuria (9). Furthermore, the patient experienced an acute exacerbation of renal injury due to the short-term excessive use of analgesics, resulting in acute analgesic nephropathy. Analgesic nephropathy primarily affects the renal tubules and interstitium, with secondary involvement of the glomeruli. Drugs and their metabolites exert direct cytotoxic effects on tubular epithelial cells, leading to degeneration and necrosis, and provoke interstitial edema. Analgesics also induce renal vasoconstriction, reducing renal blood flow and glomerular perfusion, causing ischemic shrinkage of glomeruli and further impairment of filtration. Clinically, this manifests as elevated serum creatinine and typical findings of acute kidney injury on imaging and pathology, culminating in acute deterioration of renal function.

The patient was diagnosed with SAPHO syndrome complicated by membranous nephropathy and analgesic nephropathy, necessitating a treatment strategy addressing all three conditions simultaneously. Analgesics were discontinued, and to minimize the risk of infection and drug-induced gonadotoxicity (13), a regimen of corticosteroids combined with mycophenolate mofetil (MMF) was initiated. Given the presence of fever, elevated inflammatory markers, and the imminent initiation of potent immunosuppressive therapy, short-term empirical cefuroxime was administered early to prophylactically control potential infections. During long-term follow-up, the patient demonstrated effective disease remission.

This patient’s disease course spanned seven years, with diverse and insidious symptoms, periods of undertreatment and mismanagement, and acute or subacute exacerbations, resulting in multiple transfers across hospitals and departments, thereby increasing the physical, psychological, and financial burden. These findings underscore that SAPHO syndrome is a multi-system autoimmune disorder requiring comprehensive assessment of symptoms, vigilance for early warning signs, timely completion of relevant investigations, and prompt intervention. Management should rely on multidisciplinary collaboration across rheumatology, nephrology, dermatology, neurology, radiology, and pathology to develop a comprehensive, precise, and individualized therapeutic strategy.

## Data Availability

The original contributions presented in the study are included in the article/supplementary material. Further inquiries can be directed to the corresponding authors.
